# 

**Published:** 1893-10-28

**Authors:** 


					E hospital. \ October 28a
? PRICE TWOPENCE. igeSf^ WW Ism, VilTH ?XI"RA NURSING supplemei
T" ? Bsaawssut
, transmission in the United Kingdom and Abroad.] Ditto per Aiinum, 8s., post free.
No. 370. Yol. XY. Saturday,
October 28tii, 1893.
, / [Twopence. Wgj

				

